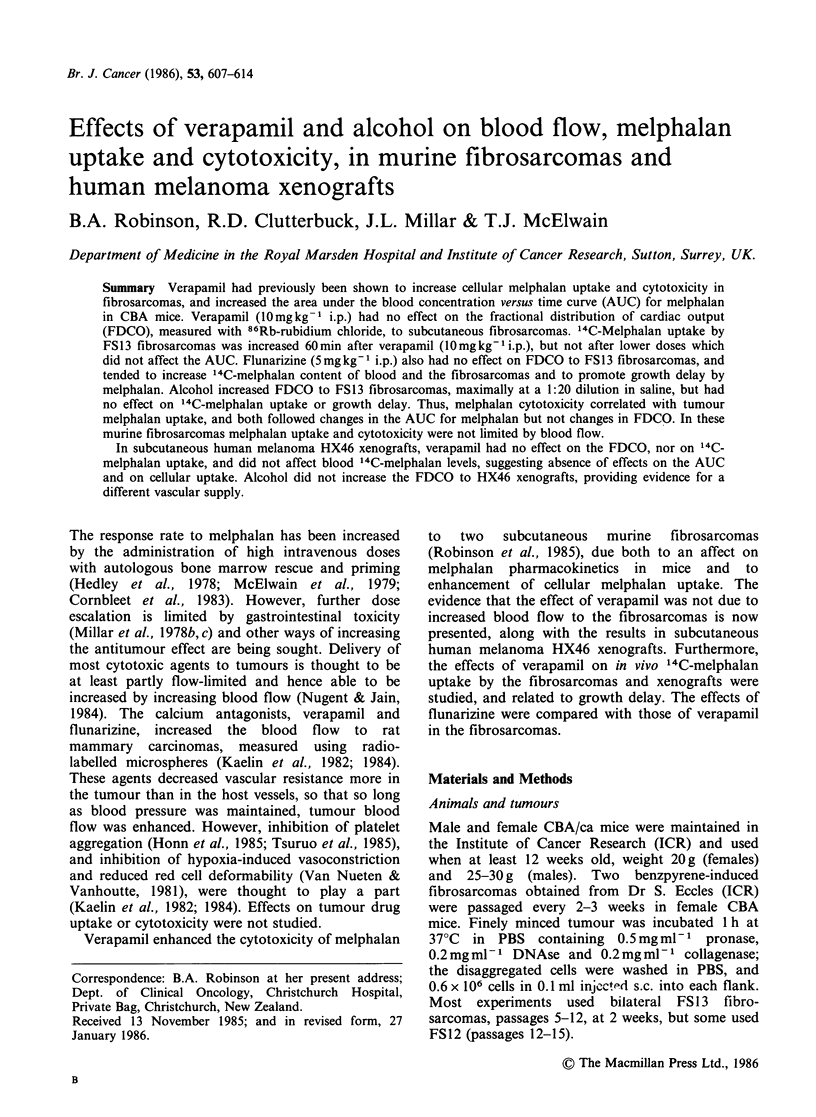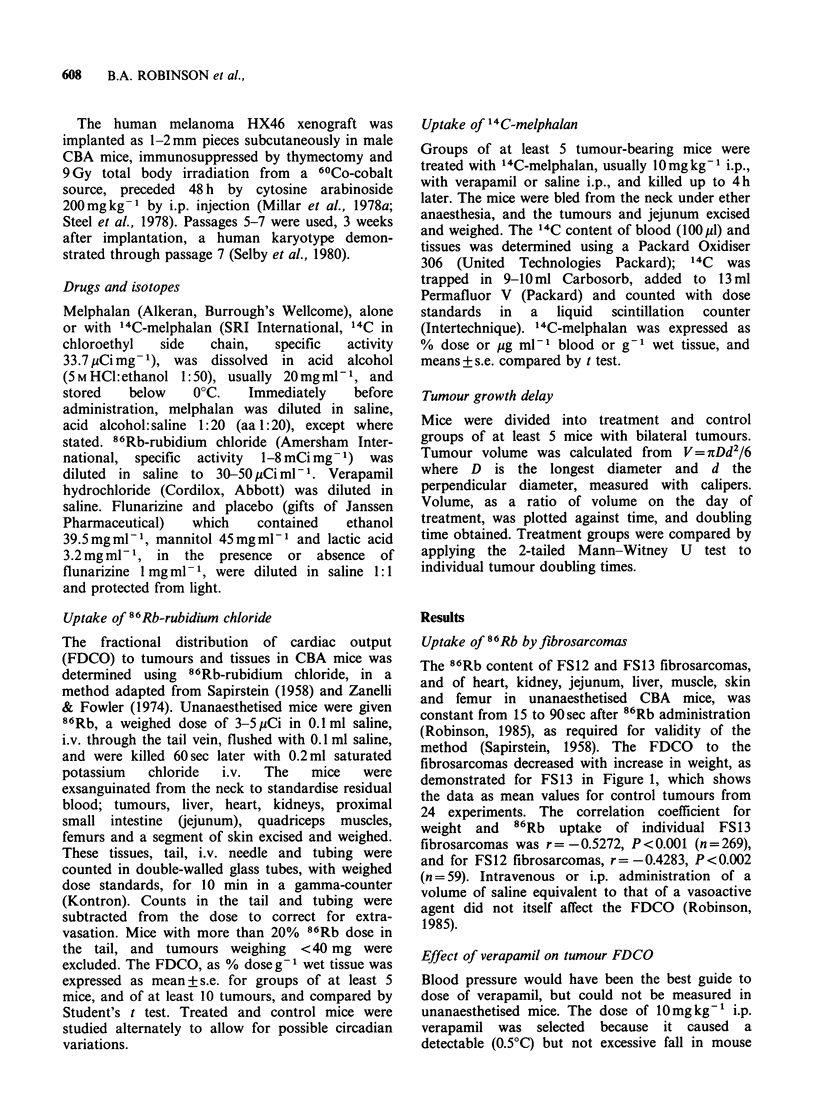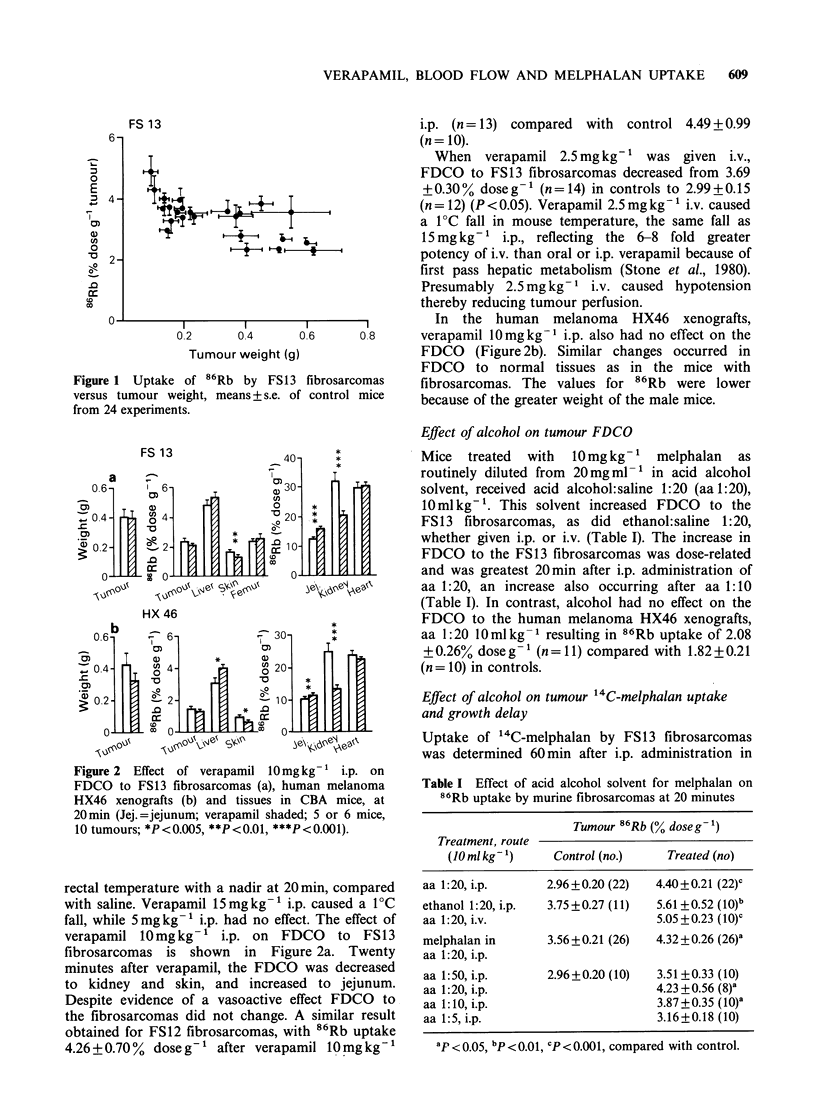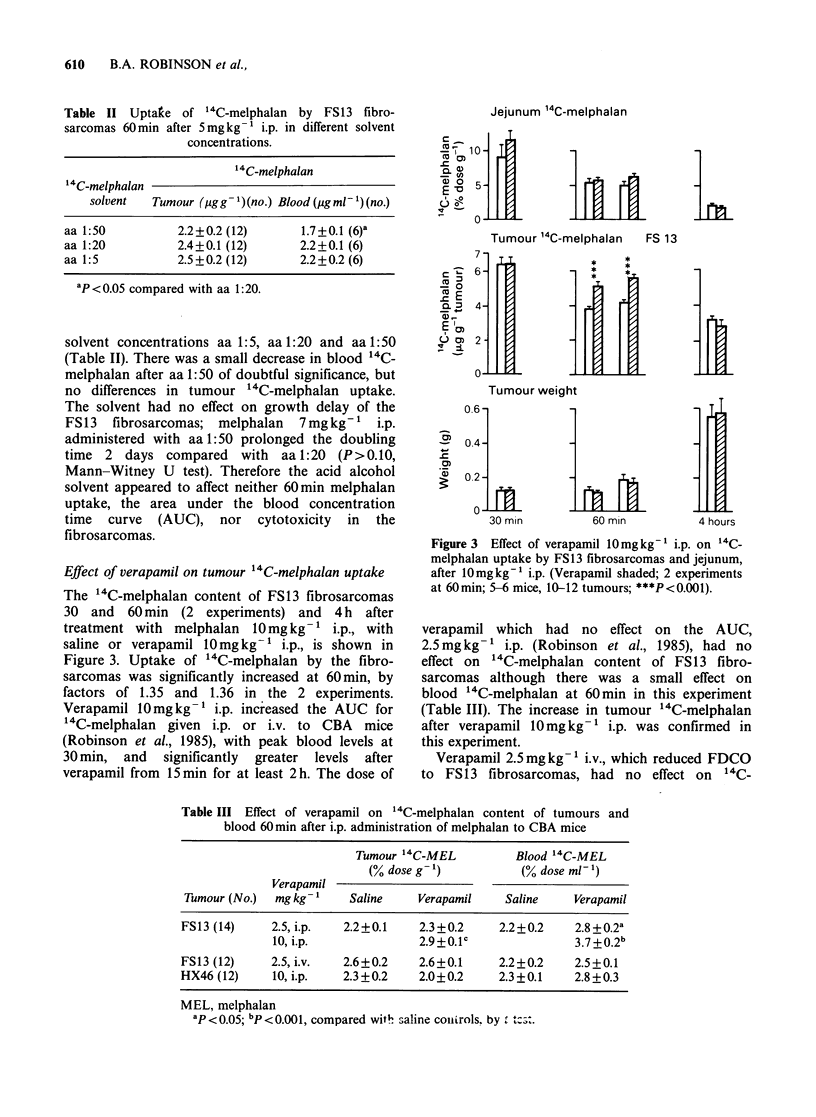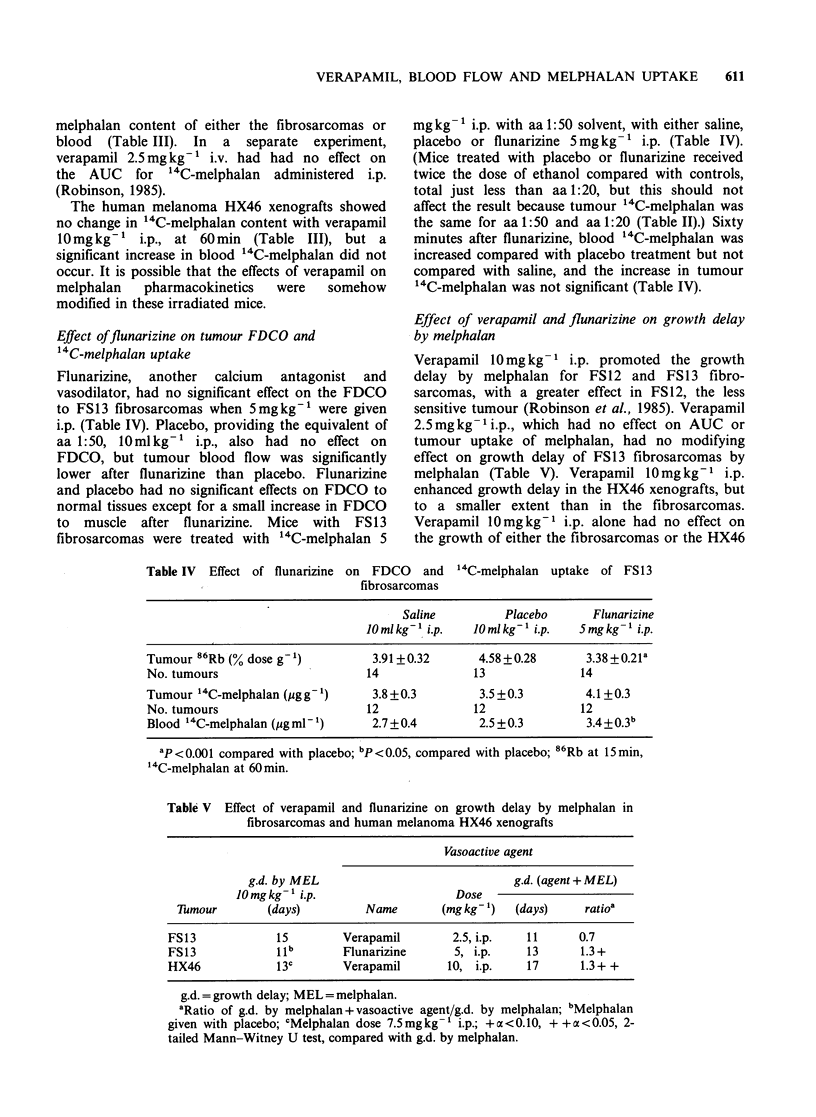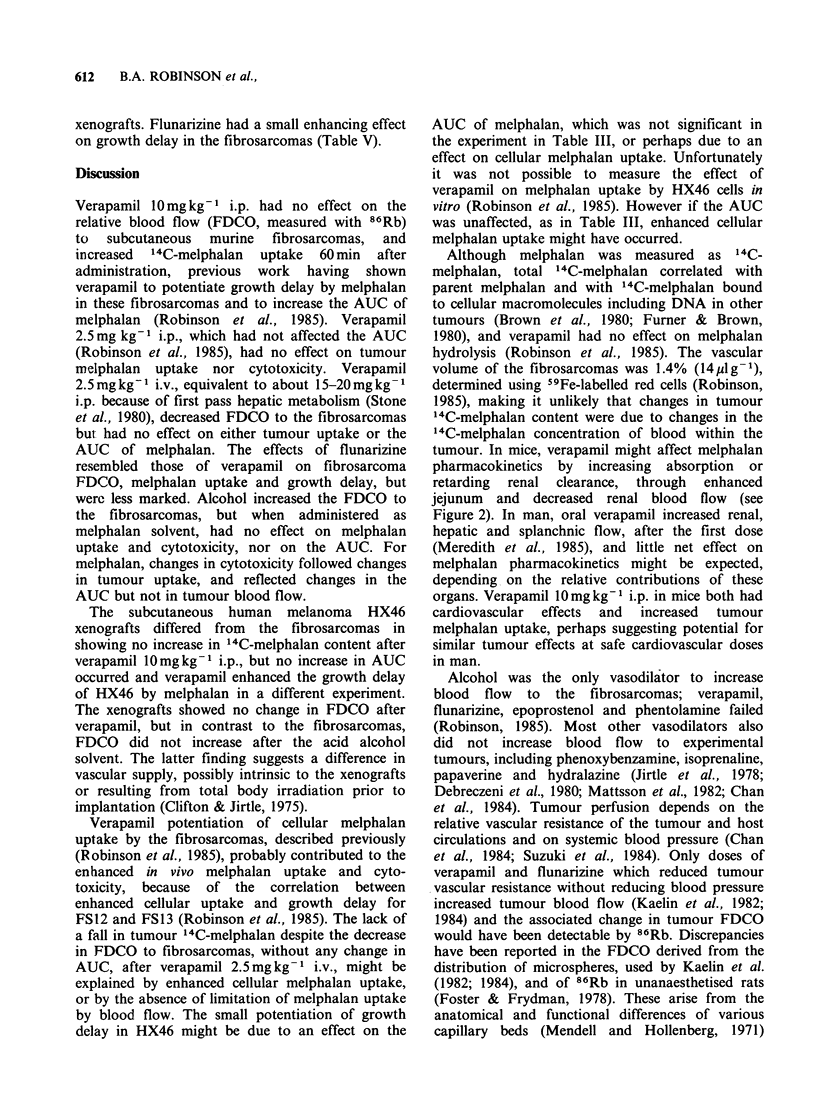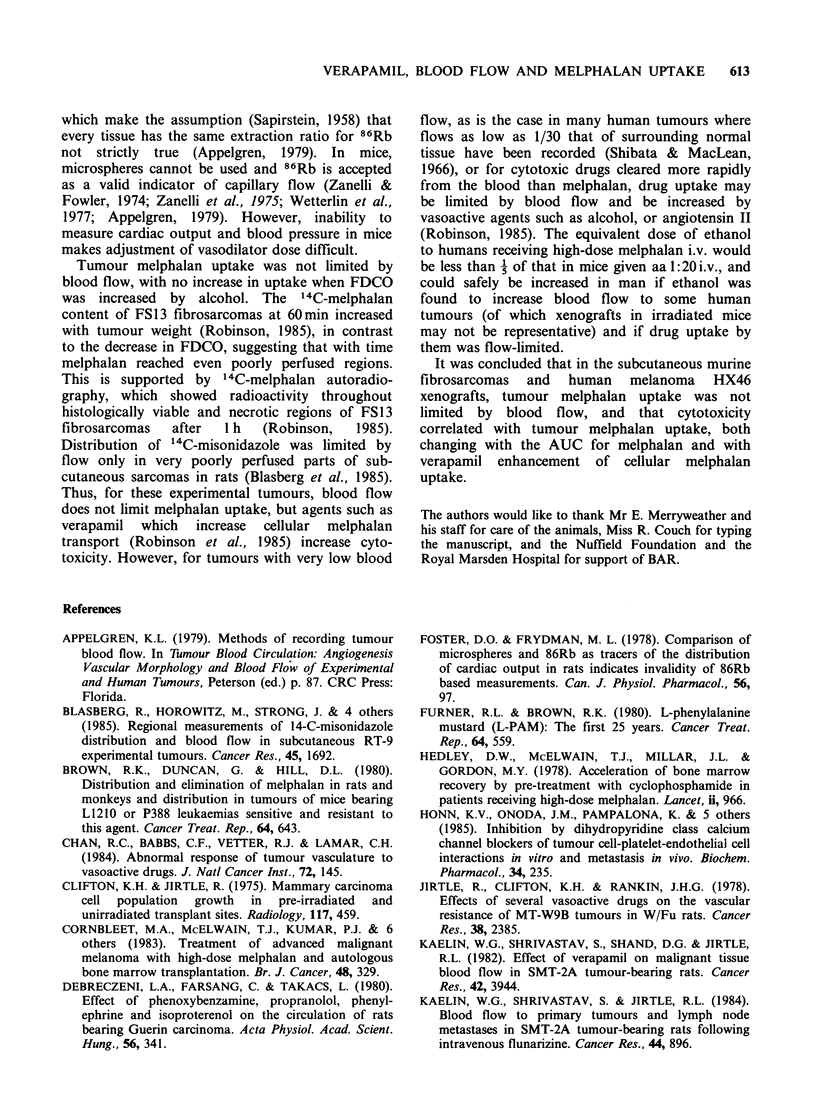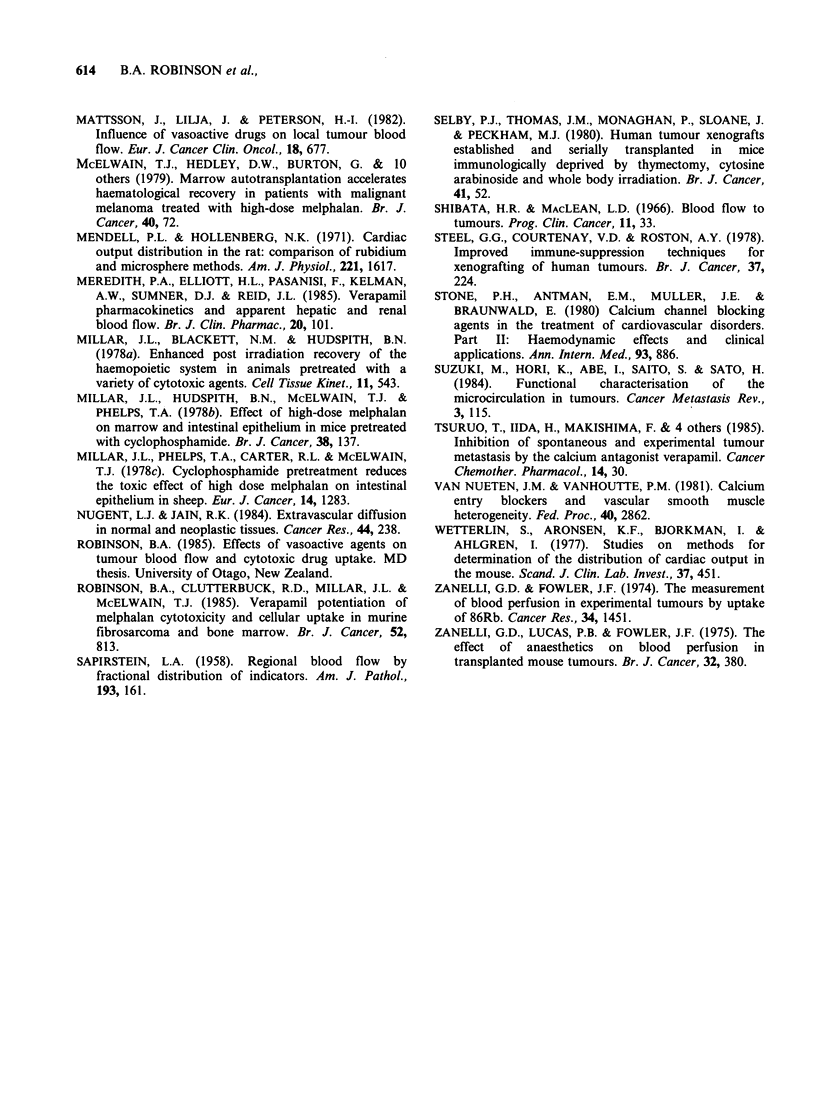# Effects of verapamil and alcohol on blood flow, melphalan uptake and cytotoxicity, in murine fibrosarcomas and human melanoma xenografts.

**DOI:** 10.1038/bjc.1986.103

**Published:** 1986-05

**Authors:** B. A. Robinson, R. D. Clutterbuck, J. L. Millar, T. J. McElwain

## Abstract

Verapamil had previously been shown to increase cellular melphalan uptake and cytotoxicity in fibrosarcomas, and increased the area under the blood concentration versus time curve (AUC) for melphalan in CBA mice. Verapamil (10 mg kg-1 i.p.) had no effect on the fractional distribution of cardiac output (FDCO), measured with 86Rb-rubidium chloride, to subcutaneous fibrosarcomas. 14C-Melphalan uptake by FS13 fibrosarcomas was increased 60 min after verapamil (10 mg kg-1 i.p.), but not after lower doses which did not affect the AUC. Flunarizine (5 mg kg-1 i.p.) also had no effect on FDCO to FS13 fibrosarcomas, and tended to increase 14C-melphalan content of blood and the fibrosarcomas and to promote growth delay by melphalan. Alcohol increased FDCO to FS13 fibrosarcomas, maximally at a 1:20 dilution in saline, but had no effect on 14C-melphalan uptake or growth delay. Thus, melphalan cytotoxicity correlated with tumour melphalan uptake, and both followed changes in the AUC for melphalan but not changes in FDCO. In these murine fibrosarcomas melphalan uptake and cytotoxicity were not limited by blood flow. In subcutaneous human melanoma HX46 xenografts, verapamil had no effect on the FDCO, nor on 14C-melphalan uptake, and did not affect blood 14C-melphalan levels, suggesting absence of effects on the AUC and on cellular uptake. Alcohol did not increase the FDCO to HX46 xenografts, providing evidence for a different vascular supply.


Br. J. Cancer (1986), 53, 607–614

### Effects of verapamil and alcohol on blood flow, melphalan uptake and cytotoxicity, in murine fibrosarcomas and human melanoma xenografts

B.A. Robinson, R.D. Clutterbuck, J.L. Millar & T.J. McElwain

*Department of Medicine in the Royal Marsden Hospital and Institute of Cancer Research, Sutton, Surrey, UK.*

**Summary** Verapamil had previously been shown to increase cellular melphalan uptake and cytotoxicity in fibrosarcomas, and increased the area under the blood concentration *versus* time curve (AUC) for melphalan in CBA mice. Verapamil ($10\,mg\,kg^{-1}$ i.p.) had no effect on the fractional distribution of cardiac output (FDCO), measured with $^{86}$Rb-rubidium chloride, to subcutaneous fibrosarcomas. $^{14}$C-Melphalan uptake by FS13 fibrosarcomas was increased 60 min after verapamil ($10\,mg\,kg^{-1}$ i.p.), but not after lower doses which did not affect the AUC. Flunarizine ($5\,mg\,kg^{-1}$ i.p.) also had no effect on FDCO to FS13 fibrosarcomas, and tended to increase $^{14}$C-melphalan content of blood and the fibrosarcomas and to promote growth delay by melphalan. Alcohol increased FDCO to FS13 fibrosarcomas, maximally at a 1:20 dilution in saline, but had no effect on $^{14}$C-melphalan uptake or growth delay. Thus, melphalan cytotoxicity correlated with tumour melphalan uptake, and both followed changes in the AUC for melphalan but not changes in FDCO. In these murine fibrosarcomas melphalan uptake and cytotoxicity were not limited by blood flow.

In subcutaneous human melanoma HX46 xenografts, verapamil had no effect on the FDCO, nor on $^{14}$C-melphalan uptake, and did not affect blood $^{14}$C-melphalan levels, suggesting absence of effects on the AUC and on cellular uptake. Alcohol did not increase the FDCO to HX46 xenografts, providing evidence for a different vascular supply.

The response rate to melphalan has been increased by the administration of high intravenous doses with autologous bone marrow rescue and priming (Hedley *et al.*, 1978; McElwain *et al.*, 1979; Cornbleet *et al.*, 1983). However, further dose escalation is limited by gastrointestinal toxicity (Millar *et al.*, 1978*b*, *c*) and other ways of increasing the antitumour effect are being sought. Delivery of most cytotoxic agents to tumours is thought to be at least partly flow-limited and hence able to be increased by increasing blood flow (Nugent & Jain, 1984). The calcium antagonists, verapamil and flunarizine, increased the blood flow to rat mammary carcinomas, measured using radio-labelled microspheres (Kaelin *et al.*, 1982; 1984). These agents decreased vascular resistance more in the tumour than in the host vessels, so that so long as blood pressure was maintained, tumour blood flow was enhanced. However, inhibition of platelet aggregation (Honn *et al.*, 1985; Tsuruo *et al.*, 1985), and inhibition of hypoxia-induced vasoconstriction and reduced red cell deformability (Van Nueten & Vanhoutte, 1981), were thought to play a part (Kaelin *et al.*, 1982; 1984). Effects on tumour drug uptake or cytotoxicity were not studied.

Verapamil enhanced the cytotoxicity of melphalan to two subcutaneous murine fibrosarcomas (Robinson *et al.*, 1985), due both to an affect on melphalan pharmacokinetics in mice and to enhancement of cellular melphalan uptake. The evidence that the effect of verapamil was not due to increased blood flow to the fibrosarcomas is now presented, along with the results in subcutaneous human melanoma HX46 xenografts. Furthermore, the effects of verapamil on *in vivo* $^{14}$C-melphalan uptake by the fibrosarcomas and xenografts were studied, and related to growth delay. The effects of flunarizine were compared with those of verapamil in the fibrosarcomas.

Correspondence: B.A. Robinson at her present address; Dept. of Clinical Oncology, Christchurch Hospital, Private Bag, Christchurch, New Zealand.
Received 13 November 1985; and in revised form, 27 January 1986.

### Materials and Methods

### *Animals and tumours*

Male and female CBA/ca mice were maintained in the Institute of Cancer Research (ICR) and used when at least 12 weeks old, weight 20 g (females) and 25–30 g (males). Two benzpyrene-induced fibrosarcomas obtained from Dr S. Eccles (ICR) were passaged every 2–3 weeks in female CBA mice. Finely minced tumour was incubated 1 h at 37°C in PBS containing $0.5\,mg\,ml^{-1}$ pronase, $0.2\,mg\,ml^{-1}$ DNAse and $0.2\,mg\,ml^{-1}$ collagenase; the disaggregated cells were washed in PBS, and $0.6 \times 10^6$ cells in 0.1 ml injected s.c. into each flank. Most experiments used bilateral FS13 fibrosarcomas, passages 5–12, at 2 weeks, but some used FS12 (passages 12–15).

© The Macmillan Press Ltd., 1986

The human melanoma HX46 xenograft was implanted as 1–2 mm pieces subcutaneously in male CBA mice, immunosuppressed by thymectomy and 9 Gy total body irradiation from a $^{60}$Co-cobalt source, preceded 48 h by cytosine arabinoside 200 mg kg$^{-1}$ by i.p. injection (Millar *et al.*, 1978*a*; Steel *et al.*, 1978). Passages 5–7 were used, 3 weeks after implantation, a human karyotype demonstrated through passage 7 (Selby *et al.*, 1980).

*Drugs and isotopes*

Melphalan (Alkeran, Burrough's Wellcome), alone or with $^{14}$C-melphalan (SRI International, $^{14}$C in chloroethyl side chain, specific activity 33.7 $\mu$Ci mg$^{-1}$), was dissolved in acid alcohol (5 M HCl:ethanol 1:50), usually 20 mg ml$^{-1}$, and stored below 0°C. Immediately before administration, melphalan was diluted in saline, acid alcohol:saline 1:20 (aa 1:20), except where stated. $^{86}$Rb-rubidium chloride (Amersham International, specific activity 1–8 mCi mg$^{-1}$) was diluted in saline to 30–50 $\mu$Ci ml$^{-1}$. Verapamil hydrochloride (Cordilox, Abbott) was diluted in saline. Flunarizine and placebo (gifts of Janssen Pharmaceutical) which contained ethanol 39.5 mg ml$^{-1}$, mannitol 45 mg ml$^{-1}$ and lactic acid 3.2 mg ml$^{-1}$, in the presence or absence of flunarizine 1 mg ml$^{-1}$, were diluted in saline 1:1 and protected from light.

*Uptake of $^{86}$Rb-rubidium chloride*

The fractional distribution of cardiac output (FDCO) to tumours and tissues in CBA mice was determined using $^{86}$Rb-rubidium chloride, in a method adapted from Sapirstein (1958) and Zanelli & Fowler (1974). Unanaesthetised mice were given $^{86}$Rb, a weighed dose of 3–5 $\mu$Ci in 0.1 ml saline, i.v. through the tail vein, flushed with 0.1 ml saline, and were killed 60 sec later with 0.2 ml saturated potassium chloride i.v. The mice were exsanguinated from the neck to standardise residual blood; tumours, liver, heart, kidneys, proximal small intestine (jejunum), quadriceps muscles, femurs and a segment of skin excised and weighed. These tissues, tail, i.v. needle and tubing were counted in double-walled glass tubes, with weighed dose standards, for 10 min in a gamma-counter (Kontron). Counts in the tail and tubing were subtracted from the dose to correct for extravasation. Mice with more than 20% $^{86}$Rb dose in the tail, and tumours weighing $<40$ mg were excluded. The FDCO, as % dose g$^{-1}$ wet tissue was expressed as mean $\pm$ s.e. for groups of at least 5 mice, and of at least 10 tumours, and compared by Student's $t$ test. Treated and control mice were studied alternately to allow for possible circadian variations.

*Uptake of $^{14}$C-melphalan*

Groups of at least 5 tumour-bearing mice were treated with $^{14}$C-melphalan, usually 10 mg kg$^{-1}$ i.p., with verapamil or saline i.p., and killed up to 4 h later. The mice were bled from the neck under ether anaesthesia, and the tumours and jejunum excised and weighed. The $^{14}$C content of blood (100 $\mu$l) and tissues was determined using a Packard Oxidiser 306 (United Technologies Packard); $^{14}$C was trapped in 9–10 ml Carbosorb, added to 13 ml Permafluor V (Packard) and counted with dose standards in a liquid scintillation counter (Intertechnique). $^{14}$C-melphalan was expressed as % dose or $\mu$g ml$^{-1}$ blood or g$^{-1}$ wet tissue, and means $\pm$ s.e. compared by $t$ test.

*Tumour growth delay*

Mice were divided into treatment and control groups of at least 5 mice with bilateral tumours. Tumour volume was calculated from $V = \pi Dd^2/6$ where $D$ is the longest diameter and $d$ the perpendicular diameter, measured with calipers. Volume, as a ratio of volume on the day of treatment, was plotted against time, and doubling time obtained. Treatment groups were compared by applying the 2-tailed Mann–Witney U test to individual tumour doubling times.

### Results

*Uptake of $^{86}$Rb by fibrosarcomas*

The $^{86}$Rb content of FS12 and FS13 fibrosarcomas, and of heart, kidney, jejunum, liver, muscle, skin and femur in unanaesthetised CBA mice, was constant from 15 to 90 sec after $^{86}$Rb administration (Robinson, 1985), as required for validity of the method (Sapirstein, 1958). The FDCO to the fibrosarcomas decreased with increase in weight, as demonstrated for FS13 in Figure 1, which shows the data as mean values for control tumours from 24 experiments. The correlation coefficient for weight and $^{86}$Rb uptake of individual FS13 fibrosarcomas was $r = -0.5272$, $P < 0.001$ ($n = 269$), and for FS12 fibrosarcomas, $r = -0.4283$, $P < 0.002$ ($n = 59$). Intravenous or i.p. administration of a volume of saline equivalent to that of a vasoactive agent did not itself affect the FDCO (Robinson, 1985).

*Effect of verapamil on tumour FDCO*

Blood pressure would have been the best guide to dose of verapamil, but could not be measured in unanaesthetised mice. The dose of 10 mg kg$^{-1}$ i.p. verapamil was selected because it caused a detectable (0.5°C) but not excessive fall in mouse

**Figure 1** Uptake of $^{86}$Rb by FS13 fibrosarcomas versus tumour weight, means±s.e. of control mice from 24 experiments.

**Figure 2** Effect of verapamil 10 mg kg$^{-1}$ i.p. on FDCO to FS13 fibrosarcomas (a), human melanoma HX46 xenografts (b) and tissues in CBA mice, at 20 min (Jej.=jejunum; verapamil shaded; 5 or 6 mice, 10 tumours; *$P<0.005$, **$P<0.01$, ***$P<0.001$).

rectal temperature with a nadir at 20 min, compared with saline. Verapamil 15 mg kg$^{-1}$ i.p. caused a 1°C fall, while 5 mg kg$^{-1}$ i.p. had no effect. The effect of verapamil 10 mg kg$^{-1}$ i.p. on FDCO to FS13 fibrosarcomas is shown in Figure 2a. Twenty minutes after verapamil, the FDCO was decreased to kidney and skin, and increased to jejunum. Despite evidence of a vasoactive effect FDCO to the fibrosarcomas did not change. A similar result obtained for FS12 fibrosarcomas, with $^{86}$Rb uptake 4.26±0.70% dose g$^{-1}$ after verapamil 10 mg kg$^{-1}$ i.p. ($n=13$) compared with control 4.49±0.99 ($n=10$).

When verapamil 2.5 mg kg$^{-1}$ was given i.v., FDCO to FS13 fibrosarcomas decreased from 3.69 ±0.30% dose g$^{-1}$ ($n=14$) in controls to 2.99±0.15 ($n=12$) ($P<0.05$). Verapamil 2.5 mg kg$^{-1}$ i.v. caused a 1°C fall in mouse temperature, the same fall as 15 mg kg$^{-1}$ i.p., reflecting the 6–8 fold greater potency of i.v. than oral or i.p. verapamil because of first pass hepatic metabolism (Stone *et al.*, 1980). Presumably 2.5 mg kg$^{-1}$ i.v. caused hypotension thereby reducing tumour perfusion.

In the human melanoma HX46 xenografts, verapamil 10 mg kg$^{-1}$ i.p. also had no effect on the FDCO (Figure 2b). Similar changes occurred in FDCO to normal tissues as in the mice with fibrosarcomas. The values for $^{86}$Rb were lower because of the greater weight of the male mice.

### *Effect of alcohol on tumour FDCO*

Mice treated with 10 mg kg$^{-1}$ melphalan as routinely diluted from 20 mg ml$^{-1}$ in acid alcohol solvent, received acid alcohol:saline 1:20 (aa 1:20), 10 ml kg$^{-1}$. This solvent increased FDCO to the FS13 fibrosarcomas, as did ethanol:saline 1:20, whether given i.p. or i.v. (Table I). The increase in FDCO to the FS13 fibrosarcomas was dose-related and was greatest 20 min after i.p. administration of aa 1:20, an increase also occurring after aa 1:10 (Table I). In contrast, alcohol had no effect on the FDCO to the human melanoma HX46 xenografts, aa 1:20 10 ml kg$^{-1}$ resulting in $^{86}$Rb uptake of 2.08 ±0.26% dose g$^{-1}$ ($n=11$) compared with 1.82±0.21 ($n=10$) in controls.

### *Effect of alcohol on tumour $^{14}$C-melphalan uptake and growth delay*

Uptake of $^{14}$C-melphalan by FS13 fibrosarcomas was determined 60 min after i.p. administration in

**Table I** Effect of acid alcohol solvent for melphalan on $^{86}$Rb uptake by murine fibrosarcomas at 20 minutes

| *Treatment, route (10 ml kg$^{-1}$)* | *Tumour $^{86}$Rb (% dose g$^{-1}$)* Control (no.) | Treated (no) |
|---|---|---|
| aa 1:20, i.p. | 2.96±0.20 (22) | 4.40±0.21 (22)[c] |
| ethanol 1:20, i.p. | 3.75±0.27 (11) | 5.61±0.52 (10)[b] |
| aa 1:20, i.v. | | 5.05±0.23 (10)[c] |
| melphalan in aa 1:20, i.p. | 3.56±0.21 (26) | 4.32±0.26 (26)[a] |
| aa 1:50, i.p. | 2.96±0.20 (10) | 3.51±0.33 (10) |
| aa 1:20, i.p. | | 4.23±0.56 (8)[a] |
| aa 1:10, i.p. | | 3.87±0.35 (10)[a] |
| aa 1:5, i.p. | | 3.16±0.18 (10) |

[a]$P<0.05$, [b]$P<0.01$, [c]$P<0.001$, compared with control.

**Table II** Uptake of ${}^{14}$C-melphalan by FS13 fibrosarcomas 60 min after 5 mg kg$^{-1}$ i.p. in different solvent concentrations.

| ${}^{14}$*C-melphalan solvent* | ${}^{14}$*C-melphalan* Tumour ($\mu g\,g^{-1}$)(no.) | Blood ($\mu g\,ml^{-1}$)(no.) |
|---|---|---|
| aa 1:50 | 2.2±0.2 (12) | 1.7±0.1 (6)[a] |
| aa 1:20 | 2.4±0.1 (12) | 2.2±0.1 (6) |
| aa 1:5 | 2.5±0.2 (12) | 2.2±0.2 (6) |

[a]$P<0.05$ compared with aa 1:20.

solvent concentrations aa 1:5, aa 1:20 and aa 1:50 (Table II). There was a small decrease in blood ${}^{14}$C-melphalan after aa 1:50 of doubtful significance, but no differences in tumour ${}^{14}$C-melphalan uptake. The solvent had no effect on growth delay of the FS13 fibrosarcomas; melphalan 7 mg kg$^{-1}$ i.p. administered with aa 1:50 prolonged the doubling time 2 days compared with aa 1:20 ($P>0.10$, Mann–Witney U test). Therefore the acid alcohol solvent appeared to affect neither 60 min melphalan uptake, the area under the blood concentration time curve (AUC), nor cytotoxicity in the fibrosarcomas.

### *Effect of verapamil on tumour ${}^{14}$C-melphalan uptake*

The ${}^{14}$C-melphalan content of FS13 fibrosarcomas 30 and 60 min (2 experiments) and 4 h after treatment with melphalan 10 mg kg$^{-1}$ i.p., with saline or verapamil 10 mg kg$^{-1}$ i.p., is shown in Figure 3. Uptake of ${}^{14}$C-melphalan by the fibrosarcomas was significantly increased at 60 min, by factors of 1.35 and 1.36 in the 2 experiments. Verapamil 10 mg kg$^{-1}$ i.p. increased the AUC for ${}^{14}$C-melphalan given i.p. or i.v. to CBA mice (Robinson *et al.*, 1985), with peak blood levels at 30 min, and significantly greater levels after verapamil from 15 min for at least 2 h. The dose of

**Figure 3** Effect of verapamil 10 mg kg$^{-1}$ i.p. on ${}^{14}$C-melphalan uptake by FS13 fibrosarcomas and jejunum, after 10 mg kg$^{-1}$ i.p. (Verapamil shaded; 2 experiments at 60 min; 5–6 mice, 10–12 tumours; ***$P<0.001$).

verapamil which had no effect on the AUC, 2.5 mg kg$^{-1}$ i.p. (Robinson *et al.*, 1985), had no effect on ${}^{14}$C-melphalan content of FS13 fibrosarcomas although there was a small effect on blood ${}^{14}$C-melphalan at 60 min in this experiment (Table III). The increase in tumour ${}^{14}$C-melphalan after verapamil 10 mg kg$^{-1}$ i.p. was confirmed in this experiment.

Verapamil 2.5 mg kg$^{-1}$ i.v., which reduced FDCO to FS13 fibrosarcomas, had no effect on ${}^{14}$C-

**Table III** Effect of verapamil on ${}^{14}$C-melphalan content of tumours and blood 60 min after i.p. administration of melphalan to CBA mice

| *Tumour (No.)* | *Verapamil mg kg$^{-1}$* | *Tumour ${}^{14}$C-MEL (% dose g$^{-1}$)* Saline | Verapamil | *Blood ${}^{14}$C-MEL (% dose ml$^{-1}$)* Saline | Verapamil |
|---|---|---|---|---|---|
| FS13 (14) | 2.5, i.p. | 2.2±0.1 | 2.3±0.2 | 2.2±0.2 | 2.8±0.2[a] |
| | 10, i.p. | | 2.9±0.1[c] | | 3.7±0.2[b] |
| FS13 (12) | 2.5, i.v. | 2.6±0.2 | 2.6±0.1 | 2.2±0.2 | 2.5±0.1 |
| HX46 (12) | 10, i.p. | 2.3±0.2 | 2.0±0.2 | 2.3±0.1 | 2.8±0.3 |

MEL, melphalan

[a]$P<0.05$; [b]$P<0.001$, compared with saline controls, by *t* test.

melphalan content of either the fibrosarcomas or blood (Table III). In a separate experiment, verapamil 2.5 mg kg$^{-1}$ i.v. had had no effect on the AUC for $^{14}$C-melphalan administered i.p. (Robinson, 1985).

The human melanoma HX46 xenografts showed no change in $^{14}$C-melphalan content with verapamil 10 mg kg$^{-1}$ i.p., at 60 min (Table III), but a significant increase in blood $^{14}$C-melphalan did not occur. It is possible that the effects of verapamil on melphalan pharmacokinetics were somehow modified in these irradiated mice.

*Effect of flunarizine on tumour FDCO and $^{14}$C-melphalan uptake*

Flunarizine, another calcium antagonist and vasodilator, had no significant effect on the FDCO to FS13 fibrosarcomas when 5 mg kg$^{-1}$ were given i.p. (Table IV). Placebo, providing the equivalent of aa 1:50, 10 ml kg$^{-1}$ i.p., also had no effect on FDCO, but tumour blood flow was significantly lower after flunarizine than placebo. Flunarizine and placebo had no significant effects on FDCO to normal tissues except for a small increase in FDCO to muscle after flunarizine. Mice with FS13 fibrosarcomas were treated with $^{14}$C-melphalan 5 mg kg$^{-1}$ i.p. with aa 1:50 solvent, with either saline, placebo or flunarizine 5 mg kg$^{-1}$ i.p. (Table IV). (Mice treated with placebo or flunarizine received twice the dose of ethanol compared with controls, total just less than aa 1:20, but this should not affect the result because tumour $^{14}$C-melphalan was the same for aa 1:50 and aa 1:20 (Table II).) Sixty minutes after flunarizine, blood $^{14}$C-melphalan was increased compared with placebo treatment but not compared with saline, and the increase in tumour $^{14}$C-melphalan was not significant (Table IV).

*Effect of verapamil and flunarizine on growth delay by melphalan*

Verapamil 10 mg kg$^{-1}$ i.p. promoted the growth delay by melphalan for FS12 and FS13 fibrosarcomas, with a greater effect in FS12, the less sensitive tumour (Robinson *et al.*, 1985). Verapamil 2.5 mg kg$^{-1}$ i.p., which had no effect on AUC or tumour uptake of melphalan, had no modifying effect on growth delay of FS13 fibrosarcomas by melphalan (Table V). Verapamil 10 mg kg$^{-1}$ i.p. enhanced growth delay in the HX46 xenografts, but to a smaller extent than in the fibrosarcomas. Verapamil 10 mg kg$^{-1}$ i.p. alone had no effect on the growth of either the fibrosarcomas or the HX46

**Table IV** Effect of flunarizine on FDCO and $^{14}$C-melphalan uptake of FS13 fibrosarcomas

| | *Saline 10 ml kg$^{-1}$ i.p.* | *Placebo 10 ml kg$^{-1}$ i.p.* | *Flunarizine 5 mg kg$^{-1}$ i.p.* |
|---|---|---|---|
| Tumour $^{86}$Rb (% dose g$^{-1}$) | 3.91 ± 0.32 | 4.58 ± 0.28 | 3.38 ± 0.21[a] |
| No. tumours | 14 | 13 | 14 |
| Tumour $^{14}$C-melphalan (μg g$^{-1}$) | 3.8 ± 0.3 | 3.5 ± 0.3 | 4.1 ± 0.3 |
| No. tumours | 12 | 12 | 12 |
| Blood $^{14}$C-melphalan (μg ml$^{-1}$) | 2.7 ± 0.4 | 2.5 ± 0.3 | 3.4 ± 0.3[b] |

[a]$P < 0.001$ compared with placebo; [b]$P < 0.05$, compared with placebo; $^{86}$Rb at 15 min, $^{14}$C-melphalan at 60 min.

**Table V** Effect of verapamil and flunarizine on growth delay by melphalan in fibrosarcomas and human melanoma HX46 xenografts

| | | *Vasoactive agent* | | | |
|---|---|---|---|---|---|
| | | | | *g.d. (agent + MEL)* | |
| *Tumour* | *g.d. by MEL 10 mg kg$^{-1}$ i.p. (days)* | *Name* | *Dose (mg kg$^{-1}$)* | *(days)* | *ratio*[a] |
| FS13 | 15 | Verapamil | 2.5, i.p. | 11 | 0.7 |
| FS13 | 11[b] | Flunarizine | 5, i.p. | 13 | 1.3 + |
| HX46 | 13[c] | Verapamil | 10, i.p. | 17 | 1.3 + + |

g.d. = growth delay; MEL = melphalan.

[a]Ratio of g.d. by melphalan + vasoactive agent/g.d. by melphalan; [b]Melphalan given with placebo; [c]Melphalan dose 7.5 mg kg$^{-1}$ i.p.; $+\alpha < 0.10$, $++\alpha < 0.05$, 2-tailed Mann–Witney U test, compared with g.d. by melphalan.

xenografts. Flunarizine had a small enhancing effect on growth delay in the fibrosarcomas (Table V).

### Discussion

Verapamil $10\,mg\,kg^{-1}$ i.p. had no effect on the relative blood flow (FDCO, measured with $^{86}Rb$) to subcutaneous murine fibrosarcomas, and increased $^{14}C$-melphalan uptake 60 min after administration, previous work having shown verapamil to potentiate growth delay by melphalan in these fibrosarcomas and to increase the AUC of melphalan (Robinson *et al.*, 1985). Verapamil $2.5\,mg\,kg^{-1}$ i.p., which had not affected the AUC (Robinson *et al.*, 1985), had no effect on tumour melphalan uptake nor cytotoxicity. Verapamil $2.5\,mg\,kg^{-1}$ i.v., equivalent to about $15–20\,mg\,kg^{-1}$ i.p. because of first pass hepatic metabolism (Stone *et al.*, 1980), decreased FDCO to the fibrosarcomas but had no effect on either tumour uptake or the AUC of melphalan. The effects of flunarizine resembled those of verapamil on fibrosarcoma FDCO, melphalan uptake and growth delay, but were less marked. Alcohol increased the FDCO to the fibrosarcomas, but when administered as melphalan solvent, had no effect on melphalan uptake and cytotoxicity, nor on the AUC. For melphalan, changes in cytotoxicity followed changes in tumour uptake, and reflected changes in the AUC but not in tumour blood flow.

The subcutaneous human melanoma HX46 xenografts differed from the fibrosarcomas in showing no increase in $^{14}C$-melphalan content after verapamil $10\,mg\,kg^{-1}$ i.p., but no increase in AUC occurred and verapamil enhanced the growth delay of HX46 by melphalan in a different experiment. The xenografts showed no change in FDCO after verapamil, but in contrast to the fibrosarcomas, FDCO did not increase after the acid alcohol solvent. The latter finding suggests a difference in vascular supply, possibly intrinsic to the xenografts or resulting from total body irradiation prior to implantation (Clifton & Jirtle, 1975).

Verapamil potentiation of cellular melphalan uptake by the fibrosarcomas, described previously (Robinson *et al.*, 1985), probably contributed to the enhanced *in vivo* melphalan uptake and cytotoxicity, because of the correlation between enhanced cellular uptake and growth delay for FS12 and FS13 (Robinson *et al.*, 1985). The lack of a fall in tumour $^{14}C$-melphalan despite the decrease in FDCO to fibrosarcomas, without any change in AUC, after verapamil $2.5\,mg\,kg^{-1}$ i.v., might be explained by enhanced cellular melphalan uptake, or by the absence of limitation of melphalan uptake by blood flow. The small potentiation of growth delay in HX46 might be due to an effect on the AUC of melphalan, which was not significant in the experiment in Table III, or perhaps due to an effect on cellular melphalan uptake. Unfortunately it was not possible to measure the effect of verapamil on melphalan uptake by HX46 cells *in vitro* (Robinson *et al.*, 1985). However if the AUC was unaffected, as in Table III, enhanced cellular melphalan uptake might have occurred.

Although melphalan was measured as $^{14}C$-melphalan, total $^{14}C$-melphalan correlated with parent melphalan and with $^{14}C$-melphalan bound to cellular macromolecules including DNA in other tumours (Brown *et al.*, 1980; Furner & Brown, 1980), and verapamil had no effect on melphalan hydrolysis (Robinson *et al.*, 1985). The vascular volume of the fibrosarcomas was 1.4% ($14\,\mu l\,g^{-1}$), determined using $^{59}Fe$-labelled red cells (Robinson, 1985), making it unlikely that changes in tumour $^{14}C$-melphalan content were due to changes in the $^{14}C$-melphalan concentration of blood within the tumour. In mice, verapamil might affect melphalan pharmacokinetics by increasing absorption or retarding renal clearance, through enhanced jejunum and decreased renal blood flow (see Figure 2). In man, oral verapamil increased renal, hepatic and splanchnic flow, after the first dose (Meredith *et al.*, 1985), and little net effect on melphalan pharmacokinetics might be expected, depending on the relative contributions of these organs. Verapamil $10\,mg\,kg^{-1}$ i.p. in mice both had cardiovascular effects and increased tumour melphalan uptake, perhaps suggesting potential for similar tumour effects at safe cardiovascular doses in man.

Alcohol was the only vasodilator to increase blood flow to the fibrosarcomas; verapamil, flunarizine, epoprostenol and phentolamine failed (Robinson, 1985). Most other vasodilators also did not increase blood flow to experimental tumours, including phenoxybenzamine, isoprenaline, papaverine and hydralazine (Jirtle *et al.*, 1978; Debreczeni *et al.*, 1980; Mattsson *et al.*, 1982; Chan *et al.*, 1984). Tumour perfusion depends on the relative vascular resistance of the tumour and host circulations and on systemic blood pressure (Chan *et al.*, 1984; Suzuki *et al.*, 1984). Only doses of verapamil and flunarizine which reduced tumour vascular resistance without reducing blood pressure increased tumour blood flow (Kaelin *et al.*, 1982; 1984) and the associated change in tumour FDCO would have been detectable by $^{86}Rb$. Discrepancies have been reported in the FDCO derived from the distribution of microspheres, used by Kaelin *et al.* (1982; 1984), and of $^{86}Rb$ in unanaesthetised rats (Foster & Frydman, 1978). These arise from the anatomical and functional differences of various capillary beds (Mendell and Hollenberg, 1971)

which make the assumption (Sapirstein, 1958) that every tissue has the same extraction ratio for $^{86}$Rb not strictly true (Appelgren, 1979). In mice, microspheres cannot be used and $^{86}$Rb is accepted as a valid indicator of capillary flow (Zanelli & Fowler, 1974; Zanelli *et al., 1975*; Wetterlin *et al.*, 1977; Appelgren, 1979). However, inability to measure cardiac output and blood pressure in mice makes adjustment of vasodilator dose difficult.

Tumour melphalan uptake was not limited by blood flow, with no increase in uptake when FDCO was increased by alcohol. The $^{14}$C-melphalan content of FS13 fibrosarcomas at 60 min increased with tumour weight (Robinson, 1985), in contrast to the decrease in FDCO, suggesting that with time melphalan reached even poorly perfused regions. This is supported by $^{14}$C-melphalan autoradiography, which showed radioactivity throughout histologically viable and necrotic regions of FS13 fibrosarcomas after 1 h (Robinson, 1985). Distribution of $^{14}$C-misonidazole was limited by flow only in very poorly perfused parts of subcutaneous sarcomas in rats (Blasberg *et al.*, 1985). Thus, for these experimental tumours, blood flow does not limit melphalan uptake, but agents such as verapamil which increase cellular melphalan transport (Robinson *et al.*, 1985) increase cytotoxicity. However, for tumours with very low blood flow, as is the case in many human tumours where flows as low as 1/30 that of surrounding normal tissue have been recorded (Shibata & MacLean, 1966), or for cytotoxic drugs cleared more rapidly from the blood than melphalan, drug uptake may be limited by blood flow and be increased by vasoactive agents such as alcohol, or angiotensin II (Robinson, 1985). The equivalent dose of ethanol to humans receiving high-dose melphalan i.v. would be less than $\frac{1}{5}$ of that in mice given aa 1:20 i.v., and could safely be increased in man if ethanol was found to increase blood flow to some human tumours (of which xenografts in irradiated mice may not be representative) and if drug uptake by them was flow-limited.

It was concluded that in the subcutaneous murine fibrosarcomas and human melanoma HX46 xenografts, tumour melphalan uptake was not limited by blood flow, and that cytotoxicity correlated with tumour melphalan uptake, both changing with the AUC for melphalan and with verapamil enhancement of cellular melphalan uptake.

The authors would like to thank Mr E. Merryweather and his staff for care of the animals, Miss R. Couch for typing the manuscript, and the Nuffield Foundation and the Royal Marsden Hospital for support of BAR.

**References**

APPELGREN, K.L. (1979). Methods of recording tumour blood flow. In *Tumour Blood Circulation: Angiogenesis Vascular Morphology and Blood Flow of Experimental and Human Tumours*, Peterson (ed.) p. 87. CRC Press: Florida.

BLASBERG, R., HOROWITZ, M., STRONG, J. & 4 others (1985). Regional measurements of 14-C-misonidazole distribution and blood flow in subcutaneous RT-9 experimental tumours. *Cancer Res.*, **45**, 1692.

BROWN, R.K., DUNCAN, G. & HILL, D.L. (1980). Distribution and elimination of melphalan in rats and monkeys and distribution in tumours of mice bearing L1210 or P388 leukaemias sensitive and resistant to this agent. *Cancer Treat. Rep.*, **64**, 643.

CHAN, R.C., BABBS, C.F., VETTER, R.J. & LAMAR, C.H. (1984). Abnormal response of tumour vasculature to vasoactive drugs. *J. Natl Cancer Inst.*, **72**, 145.

CLIFTON, K.H. & JIRTLE, R. (1975). Mammary carcinoma cell population growth in pre-irradiated and unirradiated transplant sites. *Radiology*, **117**, 459.

CORNBLEET, M.A., McELWAIN, T.J., KUMAR, P.J. & 6 others (1983). Treatment of advanced malignant melanoma with high-dose melphalan and autologous bone marrow transplantation. *Br. J. Cancer*, **48**, 329.

DEBRECZENI, L.A., FARSANG, C. & TAKACS, L. (1980). Effect of phenoxybenzamine, propranolol, phenylephrine and isoproterenol on the circulation of rats bearing Guerin carcinoma. *Acta Physiol. Acad. Scient. Hung.*, **56**, 341.

FOSTER, D.O. & FRYDMAN, M. L. (1978). Comparison of microspheres and 86Rb as tracers of the distribution of cardiac output in rats indicates invalidity of 86Rb based measurements. *Can. J. Physiol. Pharmacol.*, **56**, 97.

FURNER, R.L. & BROWN, R.K. (1980). L-phenylalanine mustard (L-PAM): The first 25 years. *Cancer Treat. Rep.*, **64**, 559.

HEDLEY, D.W., McELWAIN, T.J., MILLAR, J.L. & GORDON, M.Y. (1978). Acceleration of bone marrow recovery by pre-treatment with cyclophosphamide in patients receiving high-dose melphalan. *Lancet*, **ii**, 966.

HONN, K.V., ONODA, J.M., PAMPALONA, K. & 5 others (1985). Inhibition by dihydropyridine class calcium channel blockers of tumour cell-platelet-endothelial cell interactions *in vitro* and metastasis *in vivo*. *Biochem. Pharmacol.*, **34**, 235.

JIRTLE, R., CLIFTON, K.H. & RANKIN, J.H.G. (1978). Effects of several vasoactive drugs on the vascular resistance of MT-W9B tumours in W/Fu rats. *Cancer Res.*, **38**, 2385.

KAELIN, W.G., SHRIVASTAV, S., SHAND, D.G. & JIRTLE, R.L. (1982). Effect of verapamil on malignant tissue blood flow in SMT-2A tumour-bearing rats. *Cancer Res.*, **42**, 3944.

KAELIN, W.G., SHRIVASTAV, S. & JIRTLE, R.L. (1984). Blood flow to primary tumours and lymph node metastases in SMT-2A tumour-bearing rats following intravenous flunarizine. *Cancer Res.*, **44**, 896.

MATTSSON, J., LILJA, J. & PETERSON, H.-I. (1982). Influence of vasoactive drugs on local tumour blood flow. *Eur. J. Cancer Clin. Oncol.*, **18**, 677.

McELWAIN, T.J., HEDLEY, D.W., BURTON, G. & 10 others (1979). Marrow autotransplantation accelerates haematological recovery in patients with malignant melanoma treated with high-dose melphalan. *Br. J. Cancer*, **40**, 72.

MENDELL, P.L. & HOLLENBERG, N.K. (1971). Cardiac output distribution in the rat: comparison of rubidium and microsphere methods. *Am. J. Physiol.*, **221**, 1617.

MEREDITH, P.A., ELLIOTT, H.L., PASANISI, F., KELMAN, A.W., SUMNER, D.J. & REID, J.L. (1985). Verapamil pharmacokinetics and apparent hepatic and renal blood flow. *Br. J. Clin. Pharmac.*, **20**, 101.

MILLAR, J.L., BLACKETT, N.M. & HUDSPITH, B.N. (1978*a*). Enhanced post irradiation recovery of the haemopoietic system in animals pretreated with a variety of cytotoxic agents. *Cell Tissue Kinet.*, **11**, 543.

MILLAR, J.L., HUDSPITH, B.N., McELWAIN, T.J. & PHELPS, T.A. (1978*b*). Effect of high-dose melphalan on marrow and intestinal epithelium in mice pretreated with cyclophosphamide. *Br. J. Cancer*, **38**, 137.

MILLAR, J.L., PHELPS, T.A., CARTER, R.L. & McELWAIN, T.J. (1978*c*). Cyclophosphamide pretreatment reduces the toxic effect of high dose melphalan on intestinal epithelium in sheep. *Eur. J. Cancer*, **14**, 1283.

NUGENT, L.J. & JAIN, R.K. (1984). Extravascular diffusion in normal and neoplastic tissues. *Cancer Res.*, **44**, 238.

ROBINSON, B.A. (1985). Effects of vasoactive agents on tumour blood flow and cytotoxic drug uptake. MD thesis. University of Otago, New Zealand.

ROBINSON, B.A., CLUTTERBUCK, R.D., MILLAR, J.L. & McELWAIN, T.J. (1985). Verapamil potentiation of melphalan cytotoxicity and cellular uptake in murine fibrosarcoma and bone marrow. *Br. J. Cancer*, **52**, 813.

SAPIRSTEIN, L.A. (1958). Regional blood flow by fractional distribution of indicators. *Am. J. Pathol.*, **193**, 161.

SELBY, P.J., THOMAS, J.M., MONAGHAN, P., SLOANE, J. & PECKHAM, M.J. (1980). Human tumour xenografts established and serially transplanted in mice immunologically deprived by thymectomy, cytosine arabinoside and whole body irradiation. *Br. J. Cancer*, **41**, 52.

SHIBATA, H.R. & MACLEAN, L.D. (1966). Blood flow to tumours. *Prog. Clin. Cancer*, **11**, 33.

STEEL, G.G., COURTENAY, V.D. & ROSTON, A.Y. (1978). Improved immune-suppression techniques for xenografting of human tumours. *Br. J. Cancer*, **37**, 224.

STONE, P.H., ANTMAN, E.M., MULLER, J.E. & BRAUNWALD, E. (1980) Calcium channel blocking agents in the treatment of cardiovascular disorders. Part II: Haemodynamic effects and clinical applications. *Ann. Intern. Med.*, **93**, 886.

SUZUKI, M., HORI, K., ABE, I., SAITO, S. & SATO, H. (1984). Functional characterisation of the microcirculation in tumours. *Cancer Metastasis Rev.*, **3**, 115.

TSURUO, T., IIDA, H., MAKISHIMA, F. & 4 others (1985). Inhibition of spontaneous and experimental tumour metastasis by the calcium antagonist verapamil. *Cancer Chemother. Pharmacol.*, **14**, 30.

VAN NUETEN, J.M. & VANHOUTTE, P.M. (1981). Calcium entry blockers and vascular smooth muscle heterogeneity. *Fed. Proc.*, **40**, 2862.

WETTERLIN, S., ARONSEN, K.F., BJORKMAN, I. & AHLGREN, I. (1977). Studies on methods for determination of the distribution of cardiac output in the mouse. *Scand. J. Clin. Lab. Invest.*, **37**, 451.

ZANELLI, G.D. & FOWLER, J.F. (1974). The measurement of blood perfusion in experimental tumours by uptake of 86Rb. *Cancer Res.*, **34**, 1451.

ZANELLI, G.D., LUCAS, P.B. & FOWLER, J.F. (1975). The effect of anaesthetics on blood perfusion in transplanted mouse tumours. *Br. J. Cancer*, **32**, 380.